# Role of Hormones in the Regulation of RACK1 Expression as a Signaling Checkpoint in Immunosenescence

**DOI:** 10.3390/ijms18071453

**Published:** 2017-07-06

**Authors:** Marco Racchi, Erica Buoso, Melania Ronfani, Melania M. Serafini, Marilisa Galasso, Cristina Lanni, Emanuela Corsini

**Affiliations:** 1 Department of Drug Sciences, Università degli Studi di Pavia, Viale Taramelli 12/14, 27100 Pavia, Italy; buoso.erica@gmail.com (E.B.); melania.ronfani01@universitadipavia.it (M.R.); melania.serafini@iusspavia.it (M.M.S.); galassomarilisa@gmail.com (M.G.); cristina.lanni@unipv.it (C.L.); 2 Scuola Universitaria Superiore IUSS Pavia, Piazza della Vittoria 15, 27100 Pavia, Italy; 3 Laboratory of Toxicology, Department of Environmental Science and Policy, Università degli Studi di Milano, Via Balzaretti 9, 20133 Milano, Italy; emanuela.corsini@unimi.it

**Keywords:** aging, immunosenescence, signal transduction, protein kinase C, transcriptional regulation, cortisol, dehydroepiandrosterone, glucocorticoid receptors.

## Abstract

Immunosenescence defines the decline in immune function that occurs with aging. This has been associated, at least in part, with defective cellular signaling via protein kinase C (PKC) signal transduction pathways. Our data suggest reduced PKC activation and consequently reduced response to lipopolysaccharide (LPS) stimulation and cytokine release. The lack of PKC activation seems to be dependent on the reduced expression of the receptor for activated C kinase 1 (RACK1), a scaffolding protein involved in multiple signal transduction cascades. The defective expression of RACK1 may be dependent on age-related alteration of the balance between the adrenal hormones cortisol and dehydroepiandrosterone (DHEA). DHEA levels reduce with aging, while cortisol levels remain substantially unchanged, resulting in an overall increase in the cortisol:DHEA ratio. These hormonal changes are significant in the context of RACK1 expression and signaling function because DHEA administration in vivo and in vitro can restore the levels of RACK1 and the function of the PKC signaling cascade in aged animals and in human cells. In contrast, there is evidence that cortisol can act as a negative transcriptional regulator of RACK1 expression. The *rack1* gene promoter contains a glucocorticoid responsive element that is also involved in androgen signaling. Furthermore DHEA may have an indirect influence on the post-transcriptional regulation of the functions of the glucocorticoid receptor. In this review, we will examine the role of the hormonal regulation of *rack1* gene transcriptional regulation and the consequences on signaling and function in immune cells and immunosenescence.

## 1. Introduction

One of the most acknowledged consequences of aging is the reduced effectiveness of the immune system, which shows profound and age-dependent changes in the response to immunological challenges. The age-dependent decrease in immunological competence results from the progressive deterioration of both innate and adaptive immune responses [1]. Many factors contribute to this phenomenon, including stem-cell defects, thymic involution, aging of resting immune cells, replicative senescence of clonally expanding cells because of the erosion of telomere ends, defects in antigen-presenting cells, dysfunction in several signal transduction pathways, and dysregulation of the cytokine network [2]. Among these, the age-dependent decline of immune functions can be, at least in part, correlated with defective protein kinase C (PKC) signal transduction, which can be ascribed to the reduced expression of the Receptor for Activated C Kinase 1 (RACK1), a scaffold protein for different kinases and membrane receptors [3].

RACK1 binds activated PKCβII in order to stabilize its active conformation [4] and promote its translocation close to specific PKCβII substrates essential for immune cell activation, proliferation, differentiation, and survival [5]. We and others ([6] and refs within) have demonstrated that PKCβII activation plays a key role in the inflammatory response by inducing TNF-α release. An age-associated decrease in the release of TNF-α after lipopolysaccharide (LPS) stimulation was initially observed in alveolar macrophages obtained from aged rats, which produced ~50% less TNF-α than those from young rats [6]. A similar observation was also reported in human monocytes/macrophages, as well as in peripheral blood leukocytes, and was attributed to deficient PKC translocation due to an age-dependent decline in RACK1 expression [7,8]. As a consequence of the signal transduction impairment, a significant decrease in immune function, including the response to influenza vaccination [8], cell proliferation, and cytokine production was observed [6,7,9,10]. Interestingly, the same defective PKC signaling was also observed in the brain of aging animals, and it was demonstrated to be central in the impairment of memory processes [11,12,13].

The decrease in RACK1 expression is correlated with reduced secretion of dehydroepiandrosterone (DHEA) during aging [7]. Blood levels of DHEA are age-dependent and increase throughout childhood and puberty. After the age of 30, they decrease until reaching a minimum after the age of 80 [14]. This aspect is particularly relevant for the PKC signaling pathway because, in aged animals and in human cells, DHEA administration in vitro and in vivo can restore RACK1 levels, thus re-establishing a dose dependent TNF-α release after LPS stimulation [7,9]. Hence, part of the defective signaling in immune cells can be ascribed to age-related alteration of the hormonal balance.

This finding is particularly significant considering that cortisol levels remain substantially unchanged throughout an individual’s life, resulting in an overall increase in the cortisol:DHEA ratio [8]. This increase leads to an imbalance between the actions of these hormones, impairing the ability of DHEA to counter the effect of cortisol [15]. Cortisol acts as a negative regulator of RACK1, while DHEA inhibits cortisol activity, thereby promoting RACK1 expression [16]. The opposing effects of cortisol and DHEA seem to be derived, at least in part, from a complex influence on the post-transcriptional regulation of the glucocorticoid receptor (GR) [17,18].

These considerations underline the importance of exploring the role of RACK1 in the context of immunosenescence and the current knowledge of the mechanisms supporting the role of cortisol and DHEA in the regulation of RACK1 expression.

## 2. The Critical Involvement of Hormonal Balance Affecting RACK1 Expression

The endocrine system plays an important role in modulating immune function, and it is well established that the aging process is accompanied by hormonal changes characterized by an imbalance between catabolic hormones that remain stable and anabolic hormones such as DHEA that decrease with age [19]. In the elderly, a common observation is an imbalance between cortisol and DHEA, with an increase in the cortisol:DHEA ratio, mainly due to a significant reduction in the levels of DHEA [14,19].

Glucocorticoids have a wide spectrum of biological functions, which include stress resistance, the regulation of gluconeogenesis, cell proliferation, control of inflammation, and immune responses. Particularly relevant is the last property, which allows for their widespread use as therapeutic agents for acute and chronic inflammation, as well as in autoimmune disorders and in the treatment of leukemia and lymphoma [20].

As reviewed by Hazeldine et al. [21], there is significant evidence that DHEA can exert immunomodulatory effects that include the inhibition of glucocorticoid activity. There are some concerns about the applicability of rodents as a useful model, as the site of DHEA production in rodents appears to be localized to tissues such as the brain rather than in the adrenal glands, as is the case in humans. Nevertheless, studies in humans and human derived cells have demonstrated the role of DHEA in regulating human immunity ([7,8], reviewed in [21]). Although the clinical data, derived from the attempted modulation of the immune function with DHEA supplementation, is conflicting, there is still interest in the potential role of this steroid hormone in age-related immunosenescence, provided that its mechanism of action is properly elucidated.

The evidence that DHEA exerts anti-glucocorticoid properties on RACK1 is consistent with an antagonistic paradigm. For example, it has been demonstrated that RACK1 down-regulation caused by physiological cortisol concentrations (0.1 and 0.5 µM) could be counterbalanced by pre-treatment with physiological (10 nM) and pharmacological (100 nM) concentrations of DHEA. The most effective time of pre-incubation was 72 h, although significant effects could be demonstrated also at 16 h. The effect of DHEA was observed on the promoter activity, on the mRNA levels, and at protein level. The interaction between DHEA and cortisol on RACK1 was also tested in the context of a functional immune response, wherein THP-1 cells were treated with LPS in order to induce TNF-α release, and, as expected, pre-treatment with DHEA reduced the inhibitory effect of cortisol on LPS-induced TNF-α release [16].

The effect of cortisol on RACK1 expression is clearly transcriptional, and the experimental evidence suggests that the effect of DHEA on RACK1 is similarly transcriptional in nature. However, there is no clear indication of the interaction of DHEA with a nuclear receptor with canonical transcriptional activity. As the contrasting effect of DHEA on RACK1 expression cannot be explained by a direct interaction on the promoter region or with simple pharmacological antagonism, a number of different indirect mechanisms have been explored (Reviewed in [22]).

## 3. RACK1 and Its Transcriptional Regulation

### 3.1. RACK1: A Versatile Hub of Different Signaling Pathways

The human *rack1* gene is mapped to chromosome 5q35.3 in close proximity to the telomere of chromosome 5. The open reading frame of the gene is 1142 bp, and it encodes for a protein with 317 amino acids, registering as a 36 kDa protein on Sodium Docecyl Sulfate Polyacrylamide Gel Electrophoresis (SDS PAGE) gel [23,24]. RACK1 belongs to the tryptophan-aspartate repeat family (WD-repeat). It is homologous to the β subunit of G-protein (Gβ), the best-characterized WD repeat protein, and contains a seven-bladed β-propeller structure that serves as a docking site for interacting proteins [3].

RACK1 was first identified in a rat brain cDNA library screen designed to isolate anchor proteins that bound PKC in the presence of its activators, diacylglycerol, calcium, and phosphatidylserine [25,26,27]. The binding of RACK1 to promote signaling via PKC has been characterized for specific isoforms, including PKCβII [5], PKCδ [28], and PKCµ [29]. The extensive investigation of the relationship between RACK1 and its binding partners has led to the realization that RACK1 interacts with numerous proteins (mostly engaged in signaling), either directly or as a part of a larger complex in distinct cellular compartments [3,30]. Some of the signaling partners include Mitogen Activated Protein Kinase (MAPK), Jun-N-terminal Kinase (JNK), and cAMP specific phosphodiesterase PDE4D5, as well as Src kinase and integrins [22,31,32,33]. The functions supported by these interactions range from cell growth and survival to cell mobility and suggest a potential role for RACK1 in the development and spread of cancerous cells. The specific role of RACK1 in these aspects is, however, still controversial and appears to be cell, context, and stimulus dependent (see [31,34] for a review).

RACK1 has also been implicated as a ribosomal protein [35,36], suggesting an alternate mechanism via which this protein can alter gene translation and signal transduction. RACK1 is part of the small ribosomal subunit and promotes translation via the recruitment of PKC and the phosphorylation of the eIF6. In some types of cancers, RACK1’s function as ribosomal protein can promote the proliferation and survival of neoplastic cells [34]

Although much is known about RACK1 protein localization, interactions, and related functions, the mechanisms regulating its expression remain relatively unexplored.

### 3.2. The RACK1 Promoter Element and Its Transcription Factor Binding Sites

A bioinformatics analysis on the porcine *rack1* gene promoter identified a serum responsive element (SRE) controlling gene expression. In porcine cells, it was observed that RACK1 protein was transiently induced by serum growth factors. Similarly, RACK1 expression was positively stimulated by phorbol esters through the mediation of the AP1 binding site. Moreover, a site for the Nuclear Factor-κB (NF-κB)/c-rel transcription factor was identified [37] and later mapped in a mouse promoter, where it demonstrated a fundamental role in the regulation of RACK1 expression [38].

The promoter of the human *rack1*-encoding gene, previously described in DNA databases as guanine nucleotide binding protein β polypeptide 2-like 1 (GNB2L1), was studied by cloning a 2-kb region 5′ of the *rack1* human gene [20]. Analysis in silico suggested the presence of several binding sites for transcription factors and two major transcription start sites (TSS), similar to what was observed in the mouse gene [20,38]. Binding sites for transcription factors belonging to a smooth muscle/cardiomyocyte specific family were recognized. Consensus binding sites for Hand1/E47, Elk-1, and Nkx2-5, which are cardiac specific homeobox, and myogenin/NF1 factor, which is involved in muscle differentiation and growth, were specifically identified.

Similar to those found in the mouse and porcine genes, four c-Rel binding sites were identified in the human RACK1 promoter [24]. c-Rel is a member of the NF-κB transcription factor family, which has been demonstrated to have a role in neuronal plasticity and survival [39,40]. In SH-SY5Y human neuroblastoma cells, sAPPα, a soluble amyloid precursor protein (APP) fragment secreted in conditioned medium of cultured cells, human plasma, and the cerebrospinal fluid, could modulate the expression of RACK1 and the signaling activity of PKCβII through the activation of the PI3K/Akt and NF-κB pathways. sAPPα treatment induced c-Rel nuclear translocation, favoring its binding to the RACK1 promoter, which correlated with an increase in RACK1 expression [13]. These observations are particularly relevant in the context of Alzheimer's disease (AD), wherein RACK1 levels have been found to be significantly decreased in both membrane and cytosolic fractions obtained from AD brains when compared to age and post mortem matched control cases, suggesting a role for RACK1 expression in cognitive degeneration and age related conditions [41,42].

NF-κB involvement in RACK1 regulation was also demonstrated in cells of neural and immune origin using two well-known stimuli; LPS, an immune stimulus, and phorbol 12-myristate 13-acetate (PMA), a direct activator of the PKC pathway, which was previously reported to induce RACK1 protein expression [37]. Both molecules are linked, directly or indirectly, to nuclear cellular signals by means of the NF-κB pathway. The treatment of THP-1 (human monocytic cell line) and SH-SY5Y cells with either LPS or PMA resulted in a significant increase in RACK1 expression [24].

**Figure 1:**
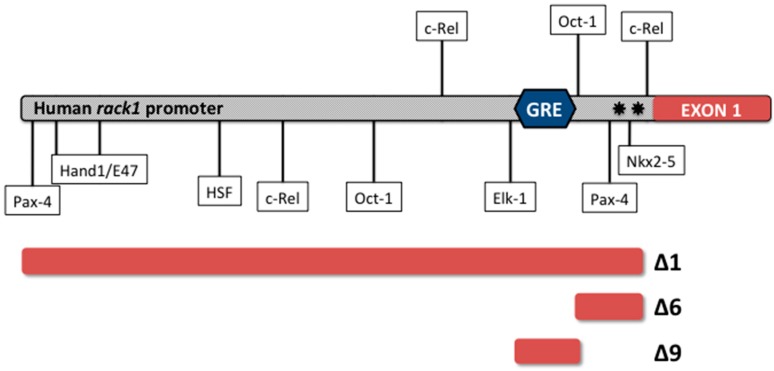
Structural analysis of the human receptor for activated C kinase 1 (RACK1) gene promoter region. Bioinformatic analysis of the 5′-flanking region within 7 kb upstream of the putative promoter region of the human *rack1* gene identified two major transcription sites, which are indicated with stars just before the beginning of Exon 1. Several putative cis-acting elements are shown; in particular, the putative unique GRE (Glucocorticoid Responsive Element) is detected at the nucleotidic position -186 with the sequence AGAACACCCTCCGGAAGCACA. Functional characterization of the GRE site was performed with deletion constructs (Δ1, Δ6, and Δ9), including or excluding the GRE site. More details can be found in the text and in [16,24].

In addition, Oct-1, Elk-1, and Pax-4 transcription factor binding sites were also identified. Finally, a consensus sequence for the binding of GR (Glucocorticoid Receptor), which appears to be similar to the consensus for a negative glucocorticoid responsive element (GRE) or nGRE, was detected at nucleotide −186 (+1 is the first TSS) [24]. nGRE binding is a new mode of sequence recognition for the human GR; two GR monomers bind nGREs in an inverted repeat orientation with strong negative cooperativity, which mediates DNA-dependent transrepression. The ability to repress to the GR at nGRE sites may allow targeted immunosuppressive therapy without the side effects often observed with glucocorticoid treatment [43]. (Figure 1).

### 3.3. RACK1 and Its Glucocorticoid Regulation

The discovery of the putative nGRE consensus sequence on the *rack1* gene promoter led to an investigation of the role of glucocorticoids in the regulation of RACK1 expression. Preliminary analyses performed in SH-SY5Y cells showed a significant repression of the activity of the *rack1* gene promoter following 24 h of treatment with 1 μM of cortisol [24]. A more detailed analysis of the role of the GRE element in controlling the RACK1 promoter was conducted in THP-1 cells transiently transfected with three luciferase reporter constructs; Δ1, Δ6, and Δ9 [16]. The Δ1 luciferase reporter construct represented the entire 2-kb region 5′ of the human *rack1* gene [24], whereas the construct Δ6 was a promoter fragment only that did not include the GRE sequence; the Δ9 construct included only the GRE sequence. THP-1 cells were transfected with these reporter constructs and treated with two physiological cortisol concentrations, 0.1 μM and 0.5 μM, which were chosen as they are representative of the most common range of the total plasma concentration of cortisol in humans. These studies demonstrated that in the presence of promoter constructs bearing the putative GCs responsive element cortisol induced a significant down-regulation of luciferase activity. In line with this evidence, cortisol was also able to drastically reduce RACK1 expression at both the mRNA and protein level, with a decline of about 70–80% compared to control cells. Additional support for a direct effect of cortisol on the promoter region of RACK1 comes from later observations that the potent GR antagonist mifepristone or RU486 abolished the cortisol-induced inhibition of luciferase activity, preventing RACK1 down-regulation [44], while the GR binding to the GRE sequence was demonstrated by ELISA based transcription factor binding assay [17]. Further evidence suggests that other corticosteroids such as betamethasone, budesonide, methylprednisolone, prednisone, and prednisolone can also target RACK1. The most effective inhibitors of LPS-induced cytokine release, namely budesonide, betamethasone, and methylprednisolone, were also most effective in reducing RACK1 mRNA expression and protein levels, thus confirming a correlation between RACK1 expression and the level of cytokine released in response to LPS. Finally, the importance of RACK1 modulation in the anti-inflammatory effect of cortisol was demonstrated using a RACK1 pseudosubstrate, which directly activates PKCβ. Cortisol inhibition of LPS-induced cytokine release was prevented when RACK1 pseudosubstrate was added together with LPS [45], suggesting that RACK1 expression is central to the anti-inflammatory effect of cortisol. Synthetic glucocorticoid recapitulated these results [44], supporting the notion that RACK1 protein is an important target of corticosteroid-induced anti-inflammatory effects. RACK1 can therefore be considered a novel transcriptional target of corticosteroid-induced anti-inflammatory effects.

## 4. DHEA and Cortisol in the Regulation of GR Isoforms

The human gene *NR3C1*, which encodes the GR, is composed of nine exons. Alternative splicing in exon 9 generates two homologous receptor isoforms, termed GRα and GRβ [46,47]. GRα mediates most of the known glucocorticoid actions, while the GRβ isoform is expressed in most tissues but lacks the ligand-binding domain. As a result, GRβ does not bind glucocorticoids and thus is unable to activate glucocorticoid-responsive gene promoters [48,49,50]. Indeed, there is evidence that GRβ acts as a dominant negative of GRα [50,51]. In the context of RACK1 expression, the presence of the GRβ /GRα inactive complex on a GRE site was demonstrated by transcription factor binding assay. When THP-1 cells were treated for 16–18 h with DHEA (10 and 100 nM) and then stimulated with cortisol (0.1 and 0.5 μM), a significant increase in the GRβ/GRα binding ratio was observed [17]. Hence, DHEA induces the increase of GRβ/GRα complex by GRβ up-regulation and counteracts the cortisol-induced binding of GRα to the RACK1 promoter region, thus reinforcing the idea that GRβ is a dominant-negative regulator of GRα activity [17]. Further investigation into the mechanism of action of DHEA in the context of GR splicing showed that DHEA induced the up-regulation of total GR mRNA, which was preferentially directed toward the β isoform, by increasing expression of the splicing factor SRSF9 (Serine/arginine Rich Splicing Factors 9), also known as SRp20 [18].

As discussed in Section 2, DHEA can modulate RACK1 protein levels via a transcriptional mechanism that does not involve a direct interaction with the promoter region of the *rack1* gene, and hence it can partially act by GRβ modulation. In line with these considerations, it was demonstrated that GRβ knockdown completely prevented DHEA-induced RACK1 expression and the modulation of cytokine release, highlighting that the effect of DHEA is driven by a modulation of GRβ expression and activity [17]. DHEA involvement in GRβ expression was confirmed by SRSF9 silencing; SRSF9 knockdown completely blocked the increase of GRβ induced by DHEA with a consequent prevention of DHEA-induced RACK1 expression [18]. These results suggest that the effect of DHEA is driven by a modulation of SRSF9, which, in turn, influences GRβ expression and activity, thus reinforcing the idea that GRβ is a dominant-negative regulator of GRα activity.

In contrast, cortisol specifically exerted a shift in the pattern of expression of the GR, promoting the α isoform at the expense of GRβ. Hence, cortisol did not affect the total GR mRNA levels, but it influenced and controlled the exon inclusion and exclusion in GR mRNA transcript by modulating, in an opposite way, SRSF3 (also known as SRp30c) and SRSF9 expression, which are two splicing factors involved in GR alternative splicing. Cortisol up-regulated SRSF3, the GRα promoting splicing factor, and down-regulated SRSF9. Moreover, cortisol-induced GRα expression was correlated with RACK1 down-regulation. In fact, SRSF3 silencing prevented the inhibitory effect of cortisol on RACK1 expression levels [18].

These data suggest that cortisol and DHEA can influence the alternative splicing of GR and underline the necessity of a critical balance between these serine/arginine-rich proteins to control the level of exon inclusion/exclusion in the mRNA transcript. Finally, these data also support the idea that the anti-glucocorticoid effect of DHEA, among other mechanisms, is also exerted by the modulation of the expression of proteins involved in the splicing of the GR pre-mRNA.

It is also worthwhile to note that the effect of DHEA on RACK1 expression could be completely prevented by using flutamide, an androgen receptor (AR) antagonist. It was demonstrated that flutamide prevented DHEA induced GRβ protein expression [52] In line with this result, DHEA-induced total GR mRNA expression was also prevented by flutamide treatment [18], according to recent evidence demonstrating an androgen response element upstream of the GR gene.

## 5. Effect of Androgens in DHEA-Induced RACK1 Expression

The physiological actions of DHEA have been attributed to its conversion to either androgens or estrogens. Recent data indicates that both THP-1 and human peripheral blood mononuclear cells (PBMCs) are able to rapidly convert DHEA to dihydrotestosterone (DHT). Hence, the ability of testosterone, DHT, and androstenedione to induce RACK1 expression and cytokine production was evaluated. As with DHEA, an increase in RACK1 expression and in LPS-induced IL-8 and TNF-α production was observed after treatment with these selected androgens. The role of DHT in DHEA-induced RACK1 expression was also corroborated by the ability of finasteride, a 5α-reductase inhibitor, to completely block the effect of DHEA on RACK1 mRNA expression. The key role of the AR to mediate DHEA-induced RACK1 expression was finally confirmed by silencing experiments [52].

Overall, these data, together with the ability of physiologically relevant concentrations of testosterone and DHT to induce RACK1 expression, support the notion that the metabolic transformation of DHEA to androgens and their binding to the AR are required for DHEA-induced RACK1 expression and cell activation.

It is important to note that approximately one-half of the AR cistrome overlaps with that of the GR. Indeed, the DNA-binding domain (DBD) of class I steroid receptors, including the AR, GR, progesterone receptor (PR), and mineralocorticoid receptor (MR), is highly conserved. All recognize a response element usually described as a canonical androgen/glucocorticoid response element (ARE/GRE) and are characterized by a well-conserved 5′-hexamer (5′-AGAACA-3′) and a less stringent sequence requirement for the 3′-hexamer [53]. In fact, different spacer-lengths or different hexamer-orientations have been proposed [54,55]. Therefore, the non-canonical GRE sequence described in the *rack1* gene promoter may also be considered as a *cis*-regulatory target of the AR, as it consists of direct repeats of the sequence 5′-AGAACAccctccggaAGCACA-3′.

In this context, and to further support the role of AR in RACK1 expression, recent data suggested a direct involvement of the AR in RACK1 regulation mediated by p,p′DDT (dichlorodiphenyltrichloroethane) and p,p′DDE (dichlorodiphenyldichloroethylene), a weak and a strong AR antagonist, respectively. In THP-1 cells transiently transfected with a luciferase reporter construct of the *rack1* gene promoter and incubated with increasing concentrations of p,p′DDT and p,p′DDE, the reporter luciferase activity was strongly reduced by both endocrine disrupting chemicals (EDC), with p,p′DDE being more potent than p,p′DDT. Moreover, the decrease in RACK1 expression was accompanied by a consequent impairment of IL-8 and TNFα release following LPS stimulation. In contrast, treatment with the AR agonist nandrolone resulted in a dose-related increase in luciferase activity and consequently in RACK1 expression. These findings suggest that RACK1 could be a relevant target of EDCs, responding in an opposing manner to agonists or antagonists of the AR and representing a bridge between the endocrine system and the innate immune system [56].

**Figure 2:**
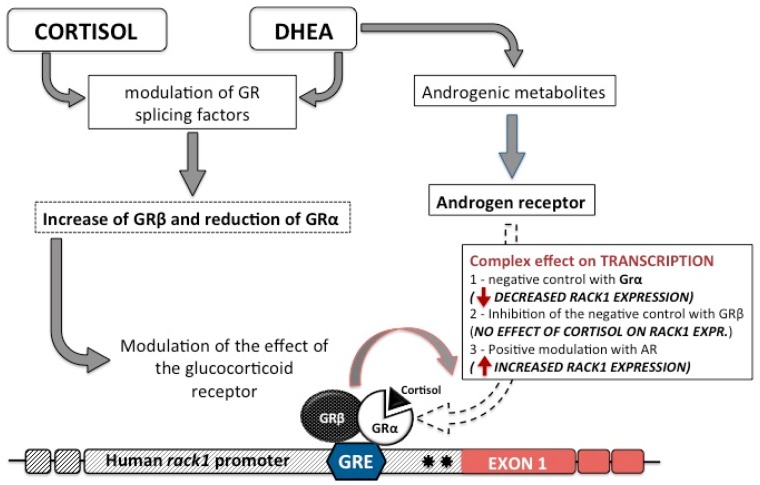
Scheme of the complex hormonal balance in the control of RACK1 expression. Data suggest that cortisol and dehydroepiandrosterone (DHEA) can influence alternative splicing of the GR, controlling the level of exon inclusion/exclusion in the mRNA transcript [17,18], and therefore suggesting that the anti-glucocorticoid effect of DHEA is due, in part, to modulation of the expression of proteins involved in the splicing of the glucocorticoid receptor (GR) pre-mRNA. In addition, the effect of DHEA on RACK1 expression is dependent on its transformation into active androgen steroids [52]. Although pharmacological evidence supports the role of the AR, there is not yet direct demonstration of the interaction of the androgen receptor (AR) with the hormone sensitive site on the *rack1* gene promoter; hence the dotted line arrow in the scheme.

These last observations should also be considered in the context of RACK1, taking into account that both the AR and GR can interact at the transcriptional level and that this interaction is correlated with their ability to form heterodimers at a common DNA site, both in vitro and in vivo. Moreover, GREs differ in their precise sequence motifs and in the functional GR surfaces required for binding or regulation. In vivo, many genomic regions that contain the GR binding sites consist only of half sites, and these regions are likely responsible for the regulation of a subset of target genes [57]. (Figure 2).

## 6. Conclusions

Taken together, these data support the existence of a complex hormonal balance between steroid hormones in the control of immune modulation, which should be further investigated within the context of immunosenescence and endocrinosenescence. A majority of the data points to a role for the cortisol:DHEA ratio in the determination of an appropriate functional response within cells of the immune system during aging. The hormonal imbalance between cortisol and DHEA observed with aging may affect directly the signal transduction cascade involved in the normal functions of key players of the innate immune system. It is therefore critical to understand the molecular mechanism through which cortisol and DHEA regulate RACK1 expression, especially considering the central role of RACK1 in cellular homeostasis. Indeed, changes in RACK1 levels are likely to subvert physiological functions, which go far beyond the immune system, possibly affecting tumor progression as demonstrated by the opposing effects of nandrolone and p,p′DDE on THP-1 cell proliferation [56].

## Abbreviations

RACK1	Receptor for Activated C Kinase1
DHEA	Dehydroepiandrosterone
PKC	Protein Kinase C
AD	Alzheimer’s disease
GRE	Glucocorticoid responsive element
AR	Androgen receptor
GR	Glucocorticoid receptor
EDC	Endocrine disrupting chemicals
LPS	Lipopolysaccharide
